# Effects of HeartWare ventricular assist device on the von Willebrand factor: results of an academic Belgian center

**DOI:** 10.1186/s12872-016-0334-z

**Published:** 2016-08-02

**Authors:** Fatemeh Esmaeilzadeh, Aurélien Wauters, Walter Wijns, Jean-François Argacha, Philippe van de Borne

**Affiliations:** 1 Department of Cardiology, Université Libre de Bruxelles (ULB), 808 Lennik Street, 1070 Brussels, Belgium; 2 Laboratory of Haemostasis, Erasme Hospital, Université Libre de Bruxelles (ULB), Brussels, Belgium; 3 Department of Cardiology, Universitair Ziekenhuis Brussel, Vrije Universiteit Brussel (VUB), Brussels, Belgium

**Keywords:** HeartWare, Left ventricle assist device, von Willebrand factor, ADAMTS13, Bleeding

## Abstract

**Background:**

Left Ventricular Assist Device (LVAD) is a promising therapy for patients with advanced heart failure (HF), but bleeding complications remain an important issue. Previous series show that acquired von Willebrand syndrome was present in up to 100 % of first generation LVAD recipients. We report the effects of new generation LVADs on vW factor (vWF) metabolism and activity in our center.

**Methods:**

Fifteen LVAD recipients (*HeartWare®, Framingham, MA, USA*) were compared to 12 HF patients, matched for age and body mass index. vWF antigen and activity, as well as D-dimers, were measured on hemostasis analyzers. A vWF LVAD-induced alteration was evocated when the [vWF activity]/[vWF antigen] ratio was <0.6. ADAMTS13 and high molecular weight multimers of vWF were also assessed.

**Results:**

LVAD recipients had similar levels of endothelial vWF production than the HF subjects (137 ± 14.5 vs. 147 ± 11.7 %; respectively, *p* = 0.611) but a decreased vWF activity (90 ± 11 vs. 132.6 ± 13 %; respectively, *p* = 0.017). [vWF activity]/[vWF antigen] ratio was 0.65 ± 0.02 in the LVAD recipients and 0.92 ± 0.06 in the subjects with HF (*p* = 0.001). ADAMTS13 activity was 80.3 ± 4.7 % in LVAD recipients and 96.2 ± 3.5 % in the HF patients (*p* = 0.016). LVAD patients disclosed markedly elevated D-dimers (3217.7 ± 735 vs. 680.6 ± 223.2 ng/mL FEU in the HF patients, *p* = 0.006). The LVAD patients experienced one major hemorrhagic event and one systemic thrombotic event during the median follow-up of 345 days.

**Conclusions:**

LVAD recipients achieved a new hemostatic equilibrium characterized by infrequent major hemorrhagic and thrombotic events, despite a mildly impaired vWF function and a markedly enhanced thrombin formation.

**Trial registration:**

ISRCTN39517567

**Electronic supplementary material:**

The online version of this article (doi:10.1186/s12872-016-0334-z) contains supplementary material, which is available to authorized users.

## Background

Patients with advanced heart failure (HF) are often older than 65 years, have multiple comorbid conditions, and as a result, are not eligible for heart transplantation [1]. Should the patient be eligible, then the lengthy waiting time until transplantation, driven by the limited organ donor availability, remains unrealistic because of the poor prognosis of the disease [2]. Left ventricular assist devices (LVADs) represent a valuable option in these patients, either as a bridge to transplantation or as a destination therapy [3]. The early pulsatile devices performed less well than the newer continuous-flow pumps, which rely on axial-flow or centrifugal flow mechanisms [4]. These devices expose the blood components to an elevated shear stress and decrease markedly pulse pressure in the arterial blood vessels.

The most frequent adverse event of LVADs is non-surgical bleeding, especially from the gastro-intestinal tract during the late follow-up period [5]. Prevalent bleeding episodes in LVAD patients have prompted lower anticoagulation targets; however other mechanisms of bleeding remain a concern. Intestinal hypoperfusion from low pulse pressure is thought to induce regional hypoxia, subsequent vascular dilation, and finally angiodysplasia [5].

Acquired von Willebrand syndrome (AvWS) also plays a role. Vascular endothelial cells express the von Willebrand factor (vWF) which assembles into multimers. These multimers bind to exposed collagen of damaged blood vessels, as well as to platelet receptors, resulting in platelet activation, adhesion, and aggregation [6]. The high molecular weight multimers of vWF (HMWM-vWF) are the most effective ones to prevent bleeding [7]. ADAMTS13 (a disintegrin and metalloproteinase with a thrombospondin type 1 motif, member 13) cleaves the vWF, under shear stress conditions in the microcirculation, at a specific site that becomes accessible when the protein unfolds. vWF conformational changes, as a result of arterial shear stress, promote vWF proteolysis by ADAMTS13, and lead to a decrease in multimer size [6]. Cleavage deficiency of vWF (due to a deficit in ADAMTS13) results in accumulation of superfluous ultra large molecular weight multimers of vWF (UL-vWF), which are prothrombotic. This is in contrast to the high shear stress encountered in the presence of a pulsatile LVAD or an aortic stenotic valve, which induce an acquired von Willebrand disease (AvWD), because the conformational changes of the HMWM-vWF resulted in excessive vWF cleavage and pathological bleedings [8]. Relief of the abnormal shear stress, as a result of cardiac transplantation in the LVAD patients, or by surgical and/or percutaneous aortic valve reopening [9] in the presence of an aortic stenosis, can correct this AvWS [8–12]. As such, HMWM-vWF decreased in patients with LVAD [10, 11], but returned to normal in 6 patients after heart transplantation, a finding already reported in an earlier cross-sectional study [12]. HMWM-vWF levels measured in random fashion in a variety of advanced HF conditions were mostly normal [11]. Moreover, in a longitudinal study, LVAD implantation decreased vWF multimers in all 37 patients [13], while, in a retrospective study, patients with centrifugal and axial flow LVADs disclosed comparable reductions in HMWM-vWF [14]. Finally, there is evidence that LVAD patients disclose an altered platelet function, which may play a role in the non-surgical bleedings. This was shown in one study where 11 out of 16 LVAD patients presented an altered ristocetin-induced platelet aggregation, which normalized in 5 subjects after heart transplantation [10].

The aim of the present study was to characterize AvWS, platelet function and bleeding events in a small group of patients implanted with third generation LVADs at the Erasme Hospital, Brussels, Belgium. These patients were compared to a group of matched HF patients. The data presented in this article were collected during a study on the effects of LVAD on micro-circulatory function [15]. As such, we have already reported that the vWF antigens did not differ between the LVAD and HF patients [15]. However no further information was provided on coagulation, since this was out of the scope of our previous manuscript [15].

## Methods

### Patients

Fifteen LVAD-supported patients were compared, using a case-control study design, to twelve male HF patients. Patient’s characteristics of 13 of these LVAD patients, and of 11 of these HF patients, were already reported in a previous study [15] . Both groups were matched for age and body mass index. The study protocol (reference: P 2013/112) was approved by the Ethical Committee of the Erasme University Hospital. A written informed consent was obtained from all patients.

### Study design

We designed a case-control study. The LVAD patients who attended the outpatient heart failure clinic were asked consecutively to participate in the study. Next, the HF patients who attended the same clinic, and whose characteristics were similar to those LVAD patients who had already participated in the study, were enrolled in the clinical trial. All abstained from meals for 8 h (except for morning medications) and from alcohol and coffee beverages for at least 12 h prior to blood collection. All were asked not to take non-steroidal anti-inflammatory drugs (NSAIDs) for at least 3 days before the blood test [15].

### Measurements and procedures

Measurements were performed by the hematology laboratory of the Erasme University Hospital (Brussels, Belgium), except for the vWF multimers and ADAMTS13 protease activity determinations (Antwerp University Hospital, Antwerp, Belgium and Saint-Luc University Hospital, Brussels, Belgium, respectively). Twenty-two mL of whole venous blood samples was collected from each patient and placed in 4 tubes of 4 mL, with 109 mmol of sodium citrate (3.2 %), and in 2 tubes of 3 mL with >15 μg/mL of hirudin.

Plasma was separated by centrifugation at 2000 *g* for 15 min at ambient temperature (20–25 °C). vWF antigen, vWF activity and D-dimers were evaluated by an immuno-turbidimetric assay, using a fully automated hemostasis analyzer (*BCS XP system, Innovance Siemens® Healthcare, USA*). LVAD-induced vWF alteration was evocated when the [vWF activity]/[vWF antigen] ratio was <0.6 [16]. Factor VIII (% activity of normal plasma) and coagulant fibrinogen (mg/dL) were determined by chronometric techniques by means of fully automated hemostasis analyzers (*BCS XP system, Siemens® Healthcare, USA; Multifibren U, Siemens® Healthcare, USA; respectively*). Prothrombin time (PT, % time of normal plasma), international normalized ratio (INR), activated Partial Thromboplastin Time (aPTT, sec) were also assessed by chronometric techniques. ADAMTS13 activity was assessed by a chromogenic ELISA method (*Technozym, Technoclone, Austria*), based on its activity on a synthetic peptide of vWF. The normal range of ADAMTS13 activity was 40–160 % of healthy subjects [17]. HMWM-vWF were studied by Western Blot analysis (*GE, Healthcare, Germany*), using SDS-agarose gel electrophoresis. Platelet aggregation was tested at physiological calcium condition by the Multiplate™ analyzer (*Dynabyte, Munich, Germany*), using agonists of thrombin receptor activating peptide-6 (TRAP-6), arachidonic acid (ASPI), adenosine diphosphate (ADP), a collagen binding activity assay (COL), and ristocetin. Ristocetin-induced platelet aggregation was determined at concentrations of 1 mg/mL.

### Clinical definition of bleeding and thrombosis

Two types of bleeding were defined prospectively: *Minor bleeding* (blood loss without transfusion) and *major bleeding* (need for transfusion >7 days after implantation, death after a bleeding, the need for re-operation, or any transfusion of packed red blood cells 7 days after implantation). Thrombosis was defined as the formation of a blood clot within one of the VAD components, or any systemic thrombo-embolic event. Medical history was retrieved from the patients’ medical records.

### Statistical analysis

All statistical analyses were performed using SPSS (*PASW Statistics 18.0, Chicago, IL, USA*). Data are expressed as mean ± SEM. All data analyses were performed in a blinded fashion in regard to the presence or absence of a LVAD. We used one-way analysis of variance (ANOVA) models, in order to determine the differences in descriptive characteristics and blood measurements among the study groups. Categorical variables were summarized by frequencies and percentages, and were analyzed by using Chi-square tests. Student *t* tests for independent samples were used to determine differences in normally distributed data. Correlation analyses using the Pearson correlation coefficient were also performed. A *p* value < 0.05 was considered statistically significant.

## Results

Patients’ characteristics are shown in Table 1 and were presented elsewhere in detail [15]. An additional dataset file shows this in more detail (see Additional file 1). Briefly, all LVAD patients had a HeartWare® Assist Device (*Framingham, MA, USA*). Continuous flow circulation was achieved with a pump speed of 2758.7 ± 62.5 rpm and a power of 4.6 ± 0.3 W. The follow up period during which events were assessed was 345 ± 63 days. The LVAD and HF groups did not differ in age, body mass index, race, history duration of heart failure and HF etiology. The LVAD group disclosed a lower proportion of men (*p* = 0.013 vs. HF patients) and was less frequently treated with angiotensin converting enzyme inhibitors or angiotensin receptor blockers (*p* = 0.030 vs. HF patients). LVAD patients received more frequently oral anticoagulant therapy (*p* = 0.001 vs. HF patients). Antiplatelet therapy prevalence did not differ, as well as the prevalence of other treatments, between both groups. Comorbidities were also similar [15].

**Table 1:** Patients’ characteristics

Parameters	LVAD (*n* = 15)	HF (*n* = 12)	*p*
Age (Y)	43 ± 4	47.2 ± 2.4	0.389
Men (%)	60	100	0.013
BMI (Kg/m^2^)	25.6 ± 1.2	29.1 ± 1.6	0.085
Race (caucasian)	14	12	0.362
Month with HF	51.3 ± 15	62 ± 20	0.665
Nonischemic HF etiology (%)	93	75	0.183
NYHA class	2.1 ± 0.1	2.2 ± 0.1	0.229
LVEF (%)	24 ± 2.6	27 ± 2.5	0.540
Heart rate (bpm)	88 ± 5	72 ± 4.6	0.030
Mean BP (mm Hg)	104 ± 11	91 ± 3.7	0.292
Medications (%)			
ß-blockers	80	100	0.100
ACE/ARB	53.3	91.7	0.030
Diuretic	80	75	0.756
Digitalic	6.7	8.3	0.869
Long acting nitrate	6.7	8.3	0.869
Statine	33.3	50	0.381
Antiarrhythmic	40	33.3	0.722
Oral anticoagulant	100	41.7	0.001
Anti-platelet therapy	66.7	50	0.381
Underlying diseases (%)			
Hypertension	6.7	41.7	0.030
Diabetes	20	16.7	0.825
Chronic renal failure	26.7	16.7	0.535
Smoker	46.7	50	0.863

*BMI* body mass index, *NYHA* New York Heart Association, *LVEF* left ventricular ejection fraction, *BP* blood pressure, *ACE*/*ARB* angiotensin converting enzyme/angiotensin receptor blocker; Diabetes (Fasting blood glucose > 126 mg/dL or antidiabetic medications); Chronic renal failure (Creatinine >2.0 mg/dL)

### Effect of LVAD on vWF profile and other blood components (Table 2, Additional file 1, Fig. 1)

**Table 2:** Blood test data

Parameters	LVAD (*n* = 15)	HF (*n* = 12)	Normal range	*p*
vWF antigen (IU/dL)	137 ± 14.5	147 ± 11.7	50–200	0.611
vWF activity (IU/dL)	90 ± 11	132.6 ± 13	50–200	0.017
[vWF activity]/[vWF antigen] ratio	0.65 ± 0.02	0.92 ± 0.06	>0.60	0.001
ADAMTS13 (%)	80.3 ± 4.7	96.2 ± 3.5	>40	0.016
Factor VIII (%)	173 ± 16	167.4 ± 16	55–205	0.813
Fibrinogen (mg/dL)	408.4 ± 23.4	371 ± 23.2	160–400	0.272
D-dimers (ng/mL FEU)	3217.7 ± 735	680.6 ± 223.2	<500	0.006
vWF multimer deficit levels	3/15	1/12	NA	0.396
Blood group “O” (% of patients)	33	41	NA	0.683
Platelet count (10^9^/L)	237 ± 25	207 ± 20	150–450	0.373
INR	2.6 ± 0.2	1.4 ± 0.2	0.8–1.2	0.001
aPTT (sec)	34.4 ± 1.2	26.5 ± 1.3	24–35	0.001
PT (%)	28.8 ± 3.5	78.2 ± 9.8	70–130 %	0.001
CRP	14.1 ± 3.8	6.45 ± 2	<10	0.108
Bilirubin total	0.58 ± 0.07	0.68 ± 0.1	<1.2	0.448
Alkaline Phosphatase	95.3 ± 15.2	77.7 ± 11.6	56–119	0.383
ALT (SGPT)	23.3 ± 3.85	56 ± 21.8	<45	0.113
AST (SGOT)	21.4 ± 1.35	47.4 ± 19.4	<35	0.146
Multiplate analysis				
Ristocetin 1 mg/mL (U)	61 ± 8.2	92.6 ± 9.7	90–201	0.019
Adenosine diphosphate (U)	62.3 ± 6.3	73.7 ± 8.7	53–122	0.285
Thrombin receptor activating peptide-6 (U)	93.7 ± 5.6	113.8 ± 6.5	94–156	0.028
Arachidonic acid (U)	33.1 ± 7.6	60.7 ± 8.5	75–136	0.024
Collagen binding activity assay (U)	43.1 ± 4.6	62.8 ± 5.8	46–117	0.012
Spontaneous (U)	10 ± 2.3	14.3 ± 2.5	1–25	0.226

*INR* international normalized ratio, *aPTT* activated partial thromboplastin time, *PT* prothrombin time, *CRP* C-Reactive Protein, *ALT* alanine aminotransferase, *AST* aspartate aminotransferase, *NA* not applicable

**Fig. 1:**
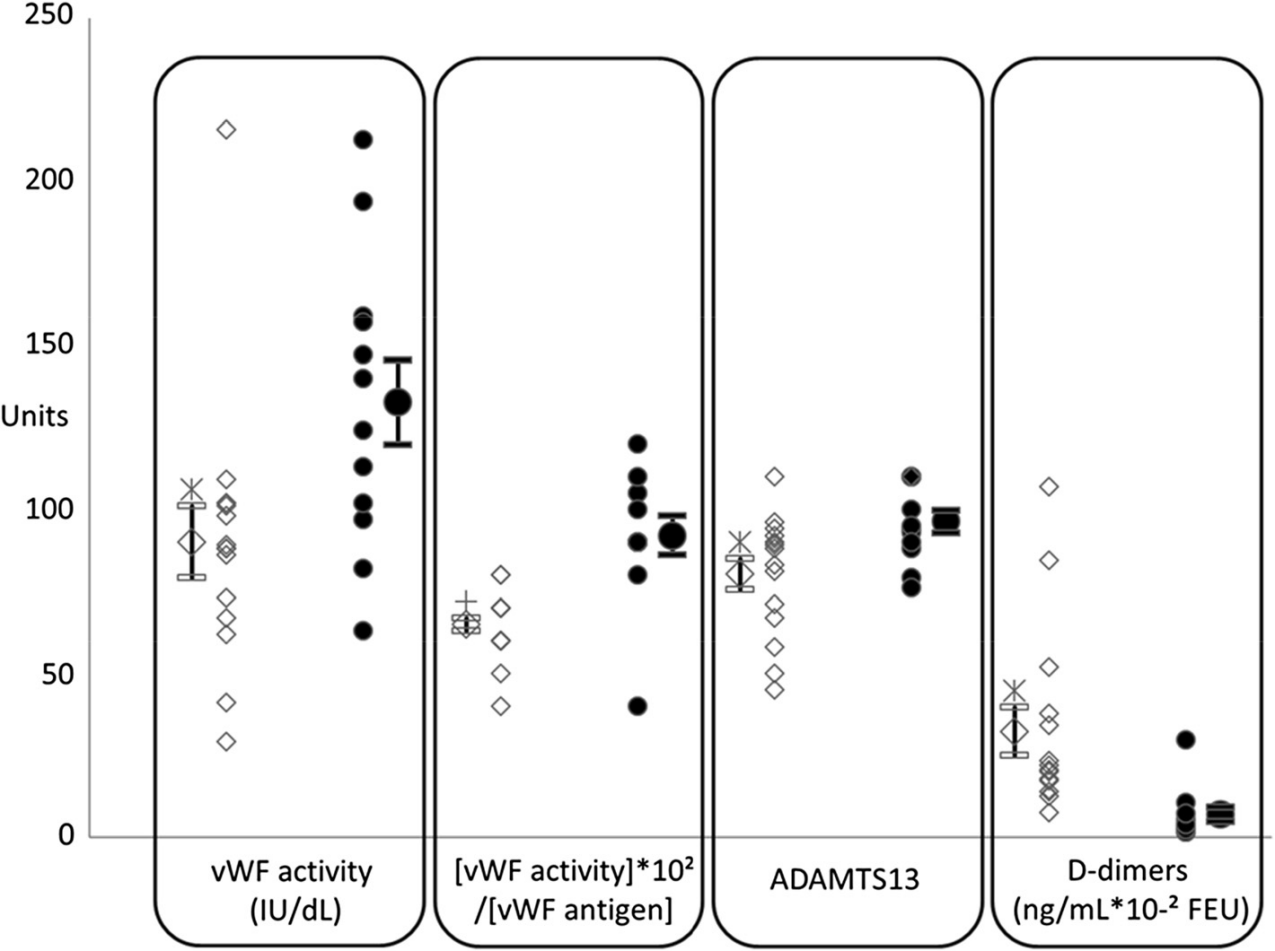
Individual measures of vWF activity (IU/dL), [vWF activity]/[vWF antigen] ratio, ADAMTS13 (%), D-dimers (ng/mL FEU) in the LVAD patients (open lozenges) and HF patients (closed circles) and their respective mean ± SEM values. _*_: *p* < 0.05, +: *p* = 0.001, number of measures seem less than actual because of overlapping individual values

Plasma vWF antigen in LVAD patients did not differ from the HF patients (*p* = 0.611). On the contrary, vWF activity was lower in the LVAD group, in comparison to the HF patients (*p* = 0.017). As a result, the [vWF activity]/[vWF antigen] ratio was reduced in the LVAD patients (*p* = 0.001 vs. HF patients). The vWF antigen and vWF activity in LVAD patients with blood group “O” did not differ from the other blood groups. ADAMTS13 activity was decreased in LVAD patients (*p* = 0.016). Factor VIII activity, fibrinogen levels, platelet count, as well as the prevalence of the “O” blood group, did not differ between the patients with or without LVADs. LVAD patients disclosed five-fold higher D-dimers levels (*p* = 0.006 vs. HF patients), higher INR and aPTT (all *p* = 0.001 vs. HF patients), while their PT was reduced (*p* = 0.001 vs. HF patients). Platelet aggregation tests in LVAD patients disclosed preserved spontaneous responses, as well as to ADP, while the responses to 1 mg/mL ristocetin, to agonists of TRAP-6, to ASPI and to COL were diminished (*p* = 0.019, 0.028, 0.024 and 0.012 respectively vs. HF patients).

### Clinical events in the LVAD patients (Additional file 1)

The LVAD recipients experienced 1 major and 4 minor bleedings during the follow-up period. One patient disclosed a spontaneous rupture of a hemangioma in the inferior pole of spleen 93 days after LVAD implantation. He received 2 packed cell units and 4 units of fresh frozen plasma (FFP). This event occurred 65 days prior participation to this study. The 4 minor bleeds consisted in 3 transient nose bleedings and 1 hemorrhoid bleeding. These 4 LVAD patients disclosed similar vWF antigen, vWF activity and [vWF activity]/[vWF antigen] ratios than those who did not experience bleeding events. The patient with a major hemorrhage presented also a clinical cerebrovascular event, unnoticeable at the brain computed tomography.

## Discussion

This study explores the effects of new generation LVADs on vWF function in patients followed in a Belgian academic centre. The main findings of our study are that our LVAD recipients presented a mild reduction in vWF activity, as compared to matched HF patients. This is consistent with previous studies in LVAD-assisted patients where vWF activity was only relatively reduced [13]. Plasma vWF antigen in LVAD patients did not differ from the HF patients, indicating similar endothelial vWF production in both groups [15]. The [vWF activity]/[vWF antigen] ratios were, on average, just above the lower limit of normalcy in the LVAD recipients, but were close to 1 in the HF patients. The fact that this ratio was lower in LVAD patients is likely due to a reduction in the HMWM. LVAD patients also disclosed five-fold elevated D-dimers levels [18], as compared to the matched HF patients, revealing a markedly enhanced thrombin formation, with subsequent risk for platelet activation and thrombotic events. This is why antiplatelet and anticoagulant therapies are recommended in LVAD patients [19]. The use of vitamin K antagonists may also indirectly influence platelet function through thrombin formation inhibition. The lower responses to TRAP-6 agonists, ASPI and COL in the LVAD recipients are most likely explained by their anti-platelet medications. However, our LVAD patients presented an altered ristocetin-induced platelet aggregation, also observed in a previous study [10]. These results might indicate that ristocetin-induced platelet aggregation is sensitive to the HMWM-vWF, since this cannot be explained by the medications taken by LVAD patients. Major hemorrhagic events were nevertheless seldom and one thrombotic event occurred during the follow-up period. Thus, our LVAD recipients were capable to achieve a remarkable new hemostatic equilibrium despite the several novel pro-hemorrhagic (such as a reduced vWF activity, antivitamin K and anti-platelet therapy) and pro-thrombotic (enhanced D-dimers formation) factors elicited by the assistance therapy.

### HMWM-vWF and ADAMTS13

The vWF antigens did not differ between the LVAD recipients and the matched HF patients. Factor VIII level, which enhances vWF proteolysis by ADAMTS13 under fluid shear stress [20], but not under static conditions, did also not differ between the LVAD and HF patients. Therefore, reductions in endothelial production of vWF and factor VIII imbalance cannot explain the mild reduction in vWF activity we observed. High shear stress, which presumably unfolds the central A2 domain of ultra large plasmatic vWF, restores the sensitivity of vWF to ADAMTS13 [21]. Pump-induced shear stress in LVAD patients might therefore enhance HMWM-vWF breakdown into smaller units, leading to lower vWF activity [22]. Elevated HMWM-vWF cleavage consumes ADAMTS13 during its action on vWF [22]. As a result, both vWF activity and ADAMTS13 levels decrease [22, 23]. This is in contrast to our HF patients, whose normal [vWF activity]/[vWF antigen] ratios were also accompanied by normal ADAMTS13 levels. Others mechanisms might nevertheless contribute to the differences in ADAMTS13 levels we observed. ADAMTS13 is predominantly synthetized in hepatic stellate cells and diffuses subsequently into capillaries and the blood stream [21]. ADAMTS13 is markedly upregulated when activated by inflammatory cytokines, such as transforming growth factor-β [24]. Moreover, experimental cholestasis and steatohepatitis elevate plasma ADAMTS13 levels [25], while partial hepatectomy in humans has the opposite effect [26]. Although not significant, alanine aminotransferase and aspartate aminotransferase were lower in the LVAD patients than in the HF patients (Table 2, Additional file 1). We cannot rule out that improved hepatic function in the LVAD patient, as compared to the non-assisted HF patients, play a role in our observations.

### Clinical events in the LVAD patients

Several studies demonstrated earlier times to first bleeding with HeartMate II (i.e. 33 days [27], 40 days [28] and 176 days after implantation [29]) as compared to the HeartWare, where the mean time to the first bleeding was 273 days [30] and 86 % of these events occurred 30 days after implantation [30]. Gastro-intestinal bleedings were reported in nearly 15.4 % of the patients supported with HeartWare [30]. In our study, the time to the first bleeding was 93 days after implantation for major bleeding, and 254 days for the minor bleedings, with a range of 87–505 days. These bleedings were not more prevalent in those assisted for a longer time. In previous studies [14, 31, 32], patients at higher centrifugal speeds demonstrated a lower percentage of HMWM-vWF and, as a result, a higher frequency of bleeding. Thus to minimize bleeding events, the LVAD speed should be the lowest possible [33, 34]. This is because low speed HeartWare therapy lessens shear stress, reduces vWF molecule unfolding and reduces ADAMTS13 accessibility to the vWF multimers. In our study, the speed of the LVAD devices in the 3 patients with low HMW-multimers was on average 218 rpm higher than in the other patients (*p* = 0.170). Nevertheless, with the low pump speeds used in our patients, bleedings were scarce despite all LVAD patients received concomitant anticoagulation therapy to prevent thrombo-embolic events. The fact that [vWF activity]/[vWF antigen] ratios remained on average just above the lower limit of normalcy in our study, may have limited the risk of bleedings in our LVAD recipients. Other studies have however shown poor relation between gastrointestinal bleedings and vWF activity [35]. This is perhaps because they did also not perform coagulation analysis at the time of bleeding. In mitigation, however, it should be remembered that the assessment of coagulation in such acute circumstances has also limitations and pitfalls [36].

**Fig. 2:**
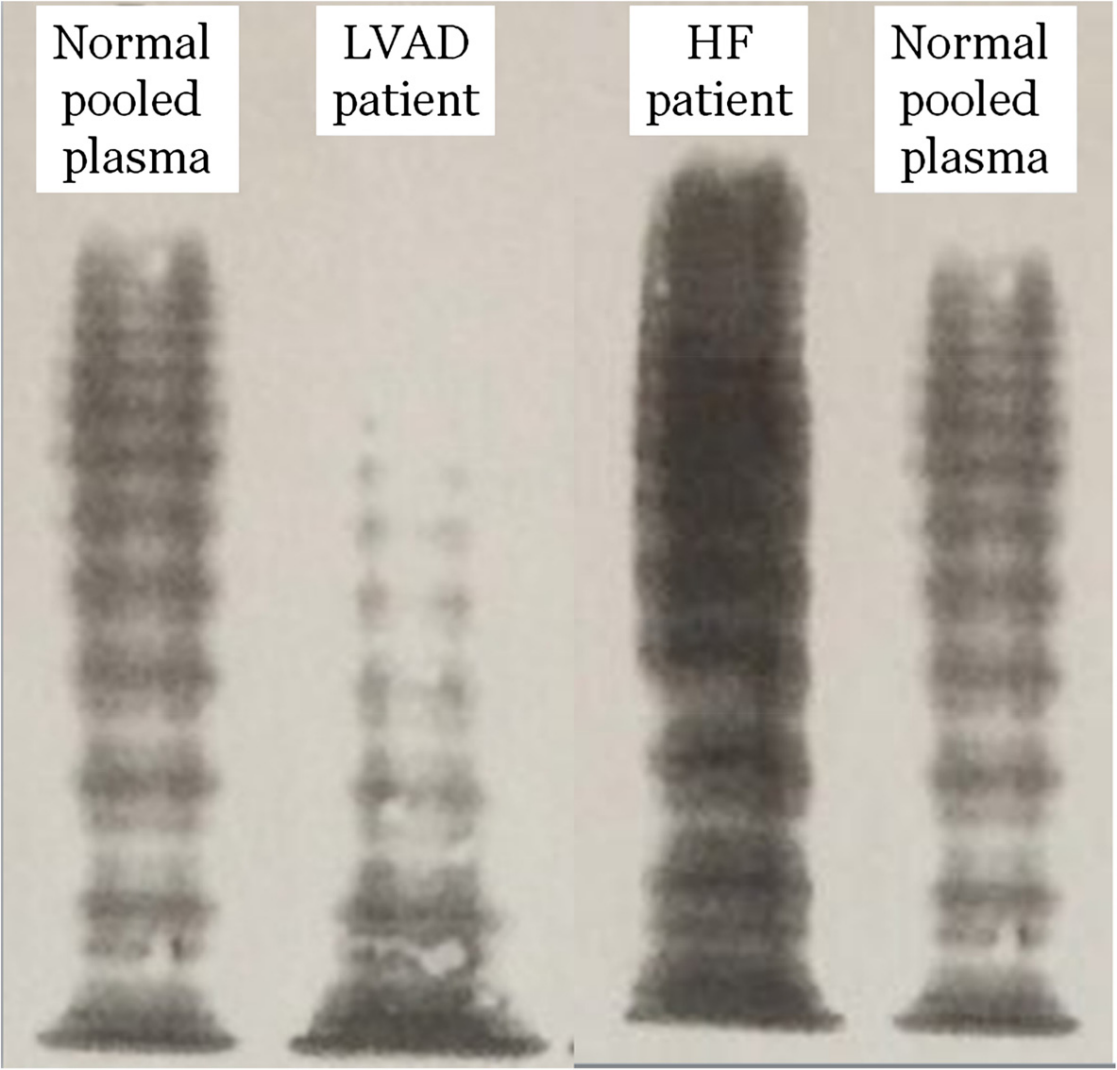
vWF multimer analysis in a low resolution gel (1.2 % SDS-agarose). The large multimers are found in the upper part of the gel. Results obtained from normal pooled plasma are compared to those from the LVAD recipients with an AvWS (presented in the Discussion section) and a HF patient with normal vWF multimers

Another limitation of our study is that we did not assess the vWF profiles before LVAD implantation. As a consequence, we cannot exclude a pre-existing AvWS prior to LVAD implantation. This was also the case in several previous publications on this topic [11, 37]. In most recent longitudinal studies, AvWS occurred in all patients after axial or centrifugal LVAD, as demonstrated by a 34 % reduction in HMWM-vWF [14]. There are reasons to believe that only one of our LVAD recipients disclosed a true AvWS. This patient had a reduced [vWF activity]/[vWF antigen] ratio of 0.44, an abnormal multimer distribution (Fig. 2) and a minor bleeding episode. His relative lower vWF activity could be due to the LVAD. However, without pre-implantation data, we cannot ascertain whether this patient had an inherited or an acquired vWS.

Four additional patients disclosed some features of a vWS. Two of these patients displayed lower HMW-multimer levels in favour of an AvWS. An inflammatory-related rise in their vWF could explain why they were nevertheless capable to maintain a normal vWF activity and did not disclose bleedings. Type 1 vWS is characterized by low levels of normally functioning vWF. This was observed in 2 patient who showed low levels of vWF activity (eventually related to blood group “O” in one, but the second one had a blood group “A”), their [vWF activity]/[vWF antigen] ratio were respectively 0.62 and 0.64 with normal multimer distribution, however they did not present bleedings.

### Limitations

In addition to the limitations already mentioned, our study has also several other pitfalls which need to be underscored, namely the small sample size of the two group of patients, the cross-sectional design of our study and the fact that the patients with LVAD could not be matched to the HF patients for gender, and inevitably, for all medication classes. Moreover, we did not characterize changes within the different vWF size ranges in our study.

## Conclusion

The recipients investigated in our study achieved a remarkable new hemostatic equilibrium despite the many novel pro-hemorrhagic and pro-thrombotic factors elicited by their LVADs. Major hemorrhagic events were seldom, and one thrombotic event occurred during the follow-up period.

## Abbreviations

ACE/ARB, angiotensin converting enzyme/angiotensin receptor blocker; ADAMTS13, a disintegrin and metalloproteinase with a thrombospondin type 1 motif, member 13; ADP, adenosine diphosphate; ALT, alanine aminotransferase; ANOVA, analysis of variance; aPTT, activated partial thromboplastin time; ASPI, arachidonic acid; AST, aspartate aminotransferase; AvWD, acquired von Willebrand disease; AvWS, acquired von Willebrand syndrome; BMI, body mass index; BP, blood pressure; COL, a collagen binding activity assay; CRP, C-reactive protein; FFP, fresh frozen plasma; HF, heart failure; HMWM-vWF, high molecular weight multimers of vWF; INR, international normalized ratio; LVAD, left ventricular assist device; LVEF, left ventricular ejection fraction; NA, not applicable; NSAIDs, non-steroidal anti-inflammatory drugs; NYHA, New York Heart Association; PT, prothrombin time; rpm, revolutions per minute; W, watt; SEM, standard error deviation; TRAP-6, thrombin receptor activating peptide-6; UL-vWF, ultra large molecular weight multimers of vWF; vWF, von Willebrand factor

## Additional file

Additional file 1: LVAD data. This file contains individual characteristics and blood tests data of the both LVAD and HF patients. (XLSX 57 kb)
